# Reduced referral and case fatality rates for severe symptomatic hyperlactataemia in a South African public sector antiretroviral programme: a retrospective observational study

**DOI:** 10.1186/1742-6405-7-13

**Published:** 2010-05-26

**Authors:** Charlotte Schutz, Andrew Boulle, Dave Stead, Kevin Rebe, Meg Osler, Graeme Meintjes

**Affiliations:** 1 Infectious Diseases Unit, GF Jooste Hospital, Duinefontein Road, Manenberg, 7764, Cape Town, South Africa; 2 Department of Medicine, Faculty of Health Sciences, University of Cape Town, Observatory, 7925, South Africa; 3 School of Public Health and Family Medicine, Faculty of Health Sciences, University of Cape Town, Observatory, 7925, South Africa; 4 Institute of Infectious Diseases and Molecular Medicine, Faculty of Health Sciences, University of Cape Town, Observatory, 7925, South Africa

## Abstract

**Background:**

Interventions to promote prevention and earlier diagnosis of severe symptomatic hyperlactataemia (SHL) were implemented in the Western Cape provincial antiretroviral programme (South Africa) from 2004. Interventions included clinician education, point-of-care lactate meters, switch from stavudine to zidovudine in high risk patients and stavudine dose reduction. This study assessed trends in referral rate, severity at presentation and case fatality rate for severe SHL.

**Methods:**

Retrospective study of severe SHL cases diagnosed at a referral facility from 1 January 2003 to 31 December 2008. Severe SHL was defined as patients with compatible symptoms and serum lactate ≥ 5 mmol/l attributable to antiretroviral therapy (ART). Cumulative ART exposure at referring ART clinics was used to calculate referral rates.

**Results:**

There were 254 severe SHL cases. The referral rate (per thousand patient years [py] ART exposure) peaked in 2005 (20.4/1000py), but fell to 1.3/1000py by 2008 (incidence rate ratio [IRR] = 0.07, 95%CI 0.04-0.11). In 2003, 66.7% of cases presented with a standard bicarbonate (SHCO_3_) level <15 mmol/l, but this fell to 12.5% by 2008 (p for trend < 0.001). Case fatality rate fell from a peak of 33.3% in 2004 to 0% in 2008 (p for trend = 0.002).

**Conclusions:**

These trends suggest the interventions were associated with reduced referral, less severe metabolic acidosis at presentation and improved survival.

## Background

Recent studies from large antiretroviral therapy (ART) cohorts in resource-limited countries have reported symptomatic hyperlactataemia (SHL) and lactic acidosis (LA) as frequent complications of ART [1-7]. The incidence of SHL/LA in sub-Saharan Africa has been reported to be between 10 - 21 per thousand patient years (py) on ART [1-3], [6] with mortality between 16 and 57% [1-6]. An important reason is that stavudine (the drug most frequently implicated as the cause of SHL and LA via mitochondrial toxicity) is used in standardized first line ART regimens in many resource limited settings due to its relative affordability.

Based on preliminary data regarding the high incidence and risk factors for SHL/LA in sub-Saharan Africa, several interventions were implemented in the Western Cape provincial ART programme in South Africa from 2004. The aim was to prevent SHL/LA and facilitate earlier diagnosis thereby improving prognosis. These interventions were: 1) clinician education and clinical guideline development on risk factors, prevention, clinical presentation, diagnosis and management (implemented from 2004) [8]; 2) distribution of point-of-care lactate meters to ART clinics from 2005 with full coverage by the end of 2006; 3) a clinical guideline advising that zidovudine rather than stavudine be used in first line ART in women with a body mass index >28 (January 2006); and 4) reduction of the stavudine dose from 40 mg twice daily to 30 mg twice daily for those > 60 kg (February 2007). The preventative switch to zidovudine was based on evidence that overweight women were at higher risk for SHL/LA [1-3], [5,9,10]. The stavudine dose reduction was based on a systematic review that showed a reduction in side effects but equivalent virological outcomes with the lower dose [11]. The point-of-care lactate meters used in primary care clinics were Roche Accutrend^® ^meters that were provided by the Western Cape provincial government and have been validated for the diagnosis of NRTI-related SHL/LA in resource limited settings[12].

We aimed to assess the impact of these preventive interventions in terms of trends in referral rate for severe SHL to our facility and, among those diagnosed with severe SHL, trends in severity of metabolic acidosis at presentation and case fatality rate.

In the Western Cape Province, ART provision in the public sector began at four pilot clinics in 2001-2 [13-15] and was followed by the wider government scale-up of ART in April 2004. Three of the four pilot clinics started with zidovudine-based ART, but by September 2003 all public sector clinics were providing a standardized first line of stavudine, lamivudine and either efavirenz or nevirapine [16]. Zidovudine was used instead of stavudine in pregnant women. GF Jooste Hospital, where the study was undertaken, is a referral hospital for all primary care ART clinics in surrounding communities. Only patients > 12 years old are seen at the hospital. There were 3 large public sector ART clinics referring to GF Jooste Hospital at the start of the study period, but this increased to 11 clinics by the end of the study period. All patients with significant ART related complications, including all with suspected SHL/LA, were referred to GF Jooste Hospital. Provincial guidelines stated all patients with clinically suspected SHL or LA and all patients with lactate > 4 mmol/l measured on point-of-care lactate meter in primary care should be referred to the appropriate secondary level hospital for assessment and laboratory lactate measurement. These guidelines were the same for all primary care ART clinics in the referral area.

A previous study from our hospital describing the clinical features of severe SHL was conducted between August 2003 and November 2005. It included 75 cases, all of whom are also included in this paper. In the earlier study, 95% of patients were female, the median age was 33 years (range 21 - 57), and median duration of ART at presentation was 10 months (interquartile range [IQR] 8 - 12). All patients were on a stavudine containing regimen or had recently switched from stavudine to zidovudine (8 patients had switched to zidovudine within the preceding 3 weeks). 71% of the patients were found to have metabolic acidosis (standard bicarbonate [SHCO_3_] < 20 mmol/l) [2].

## Methods

A retrospective observational study from 1 January 2003 to 31 December 2008 was conducted. Laboratory and patient records were reviewed and all cases with severe SHL, defined by compatible symptoms and a serum lactate of ≥5 mmol/l were included, provided the hyperlactataemia was attributable to ART. Patients were excluded if they had another acute illness that was more likely the cause of hyperlactataemia. Reasons for exclusion were severe dehydration, hepatic failure, sepsis and severe anaemia. However, if the lactate remained elevated after treatment of the acute condition, suggesting underlying NRTI-related mitochondrial toxicity, the patient was included. The study period was divided into 6 calendar years. The referral rate for severe SHL was calculated using two methods. For the first calculation, the denominator used was the cumulative ART exposure among adult patients at the referring ART clinics over that period, based on monthly reports of total patients in care in each referring clinic at the end of each month [15]. For the second calculation, the denominator used was the cumulative adult ART exposure of patients on ART for between 6-18 months duration. We performed the second calculation to correct for potential biasing effect of more patients in the denominator being beyond the "window of risk" for severe SHL in later calendar years. This "window of risk" has been reported to be between 6 and 18 months on ART from several studies conducted in resource limited settings where stavudine was used in first line ART. Most patients in these studies were on stavudine when they developed SHL or LA [1-7].

Venous blood samples for lactate were taken without a tourniquet, into a fluoride oxalate containing tube, and rapidly transported to the laboratory for processing. Lactate was measured on a Beckman-Coulter Synchron CX^® ^system. Blood gas for pH and SHCO_3 _were done on venous or arterial blood samples. Our laboratory uses a reference range of 0.6-2.45 mmol/l to define normal serum lactate. A value of 5 mmol/l was thus used to define severe SHL because this is twice the upper limit of normal. SHCO_3 _level < 15 mmol/l at presentation was used as an indicator of more severe metabolic acidosis. A previous study done at our hospital showed that SHCO_3 _level <15 mmol/l was associated with acute mortality (odds ratio = 22.5, 95% confidence interval [95% CI] 2.8-1,045.7) [2]. Univariate Poisson regression analysis was used to calculate incidence rate ratios (IRRs) for referral rates. The non-parametric test for trend across ordered groups was used to assess trends in median SHCO_3 _level at presentation. The chi-squared test for trend was used to assess trends with respect to proportions. Permission for the study was obtained from the University of Cape Town Research Ethics Committee (REC REF: 312/2005 and 431/2009).

## Results

Two hundred and fifty nine (259) cases of severe SHL presented during the study period. Folders were missing for 5, thus 254 cases were included in the analysis. Seventy five of these cases have been included in previous reports [2,10]. Two hundred and twenty three (87.8%) were female with a median age of 35 (IQR 29 - 41.5). The 31 male patients had a median age of 42 (IQR 36.5 - 46). The first case presented in August 2003. During the study period the number of adult patients on ART in our referral area increased from 268 in January 2003 to 13 985 in December 2008.

**Figure 1:**
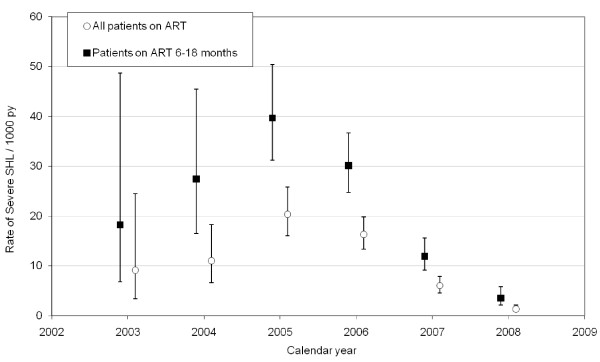
**Referral rates for severe symptomatic hyperlactataemia**. The referral rates from 2003 to 2008 are shown with 95% confidence intervals. The empty circles demonstrate referral rates using cumulative antiretroviral therapy (ART) exposure of all adult patients at referral clinics as the denominator. The solid squares demonstrate referral rates using cumulative adult ART exposure of between 6 and 18 months duration as the denominator. SHL = symptomatic hyperlactataemia; py = patient years, ART = antiretroviral therapy

Trends in referral rate are shown in Figure 1 and Table 1. The referral rate peaked in 2005, but fell significantly by 2007 and 2008. Using cumulative adult ART exposure in the referral area as the denominator and 2005 as the reference, the IRR was 0.3 (95% CI = 0.21-0.43) for 2007 and 0.07 (95% CI = 0.04-0.11) for 2008. There was a similar decrease in referral rates from 2005 to 2007 and 2008 using adult ART exposure of 6-18 months duration as the denominator. Trends in SHCO_3 _level at presentation and case fatality rate are shown in Table 2. The median SCHO_3 _at presentation increased over the study period (p for trend < 0.001) resulting in a decrease in the proportion of cases with SCHO_3 _level < 15 mmol/l at presentation (p for trend < 0.001). The case fatality rate dropped substantially from 2004 to 2008 (p for trend = 0.002).

**Table 1:** Case load and referral rates for severe symptomatic hyperlactataemia cases (2003-2008)

Time Period	Number of severe SHL cases	Cumulative adult ART exposure (years)	Referral rate: cases/1000 py ART exposure (95% CI)	IRR(95% CI)	Cumulative ART exposure 6-18 months (years)	Referral rate: cases/1000 py 6-18 months on ART (95% CI)	IRR(95%CI)
2003	4	436	9.2 (3.4-24.4)	0.45 (0.16-1.24)	219	18.3 (6.9-48.7)	0.46 (0.17-1.26)

2004	15	1361	11.0 (6.6-18.3)	0.54 (0.31-0.95)	547	27.4 (16.5-45.5)	0.69 (0.39-1.21)

2005	67	3292	20.4 (16.0-25.9)	1.0 (ref)	1689	39.7 (31.2-50.4)	1.0 (ref)

2006	99	6069	16.3 (13.4-19.9)	0.8 (0.59-1.09)	3284	30.1 (24.8-36.7)	0.76 (0.56-1.04)

2007	53	8771	6.0 (4.6-7.9)	0.3 (0.21-0.43)	4442	11.9 (9.1-15.6)	0.30 (0.21-0.43)

2008	16	12014	1.3 (0.8-2.2)	0.07 (0.04-0.11)	4488	3.6 (2.2-5.8)	0.09 (0.05-0.16)

Entire Period	254	31943	8.0 (7.0-9.0)	-	14669	17.3 (15.3-19.6)	-

^1 ^Incidence rate ratio was calculated using 2005 as reference period.

SHL = Symptomatic Hyperlactataemia, ART = Antiretroviral therapy, py = patient years, CI = Confidence interval, IRR = Incidence rate ratio.

**Table 2:** Standard bicarbonate levels and case fatality rates for severe symptomatic hyperlactataemia cases (2003-2008)

Time Period	Number of severe SHL cases	Number who had SHCO_3 _performed (%)	Median SHCO_3 _(IQR)^1^	Number with SHCO_3 _level <15 mmol/l (%)^2^	Number of deaths (case fatality rate as%)^3^
2003	4	3 (75)	14.2 (5.1-19.8)	2 (66.7)	0 (0)

2004	15	14 (93.3)	15.5 (4.9-18)	7 (50)	5 (33.3)

2005	67	55 (82.1)	17.6 (13.5-20)	19 (34.5)	7 (10.4)

2006	99	89 (89.9)	19.8 (17-21.3)	13 (14.6)	5 (5.1)

2007	53	49 (92.5)	20.1 (18-21.2)	6 (12.2)	3 (5.7)

2008	16	16 (100)	20 (17.3-21.4)	2 (12.5)	0 (0)

Entire period	254	226 (89)	19.1 (15.8-20.9)	49 (21.7)	20 (7.9)

^1 ^Median standard bicarbonate level: p for trend < 0.001 (for period 2003-2008).

^2 ^Proportion with standard bicarbonate < 15 mmol/l: p for trend < 0.001 (for period 2003-2008). The proportion was calculated using the number who had standard bicarbonate performed during that year as denominator, see column 3.

^3^Case fatality rate: p for trend = 0.002 (for period 2004 - 2008; 2003 was excluded as case fatality rate peaked in 2004).

SHL = Symptomatic Hyperlactataemia, SHCO_3 _= Standard bicarbonate in mmol/l (normal range 21-26 mmol/l), IQR = interquartile range.

## Discussion

Despite a growing number of patients on ART at the primary care clinics in the referral area of our hospital, the referral rate, severity of metabolic acidosis at presentation and case fatality rate for severe SHL fell during the study period. It is likely that the preventative switch to zidovudine in overweight women and lowering the stavudine dose led to reduced incidence which resulted in a reduced referral rate. Heightened clinician vigilance and the availability of a point-of-care test in primary care clinics likely led to earlier detection of SHL and more proactive substitutions in the primary care setting.

The proportion of females starting ART in our referral area has not changed substantially since 2003. In 2003, females accounted for 68.9% of adults starting ART in our referral area, and in 2008, 66.1% of adults starting ART were female. Female gender is a risk factor for severe SHL [1-3], [5,6], [9,10]. It is unlikely that the reduction in severe SHL referral rate that we observed was accounted for by this relatively small reduction in the proportion of females starting ART.

Inpatient management of severe SHL at our facility (which includes intravenous fluids, intravenous bicarbonate in those with severe metabolic acidosis, intravenous thiamine/vitamin B complex and empiric broad-spectrum antibiotics [2]) did not change during the study period. Specifically, there was also no change in the management of patients who were critically ill with lactic acidosis who were admitted to the high care unit throughout the study period for full supportive management which included broad-spectrum intravenous antibiotics to treat possible sepsis.

Thus the improved survival is likely attributable to earlier diagnosis, as evidenced by less severe metabolic acidosis at presentation in the later calendar years. In 2004 the mortality rate was 33.3%, which correlates with the mortality rate reported from other resource-limited settings [1-6]. The mortality rate decreased after 2004 and remained low, with no deaths due to SHL recorded in 2008.

This was a retrospective folder review, which has important limitations. We were dependant on clinical notes from attending clinicians. We also assumed that clinicians in primary care ART clinics detected and referred cases consistently throughout the study period. Any change in referral practice may have introduced selection bias. However, our experience is that referral practices have improved over time as the ART programme has matured making this an unlikely cause of the reduced referral rates we observed.

It is possible that patients with severe SHL could have died before being referred or could have died acutely in the casualty unit without collateral history and before lactate was measured. Thus we reported the referral rate rather than an incidence rate, but it is likely that the referral rate approximates the incidence rate as all cases of suspected and confirmed severe SHL are referred from primary care ART facilities to our hospital. The referral rates during 2004 and 2005, prior to the impact of the interventions, are comparable to incidence rates reported from elsewhere in sub-Saharan Africa [1-3], [6]. We did not capture detailed clinical data on the cases and are unable to report on current ART regimen, duration of ART or stavudine dose among the cases in this study.

## Conclusions

These trends suggest the measures implemented resulted in reduced incidence, earlier diagnosis and thus less severe presentations and improved survival of patients with severe SHL in our referral area. Furthermore it demonstrates how through pharmacovigilance within large public health programs, early warning signs can lead to beneficial interventions that may mitigate ART adverse events. Mitochondrial toxicity associated with stavudine, however, still causes significant morbidity in the form of neuropathy [17] and lipoatrophy in our setting and alternatives to stavudine are required.

## Competing interests

The authors declare that they have no competing interests.

## Authors' contributions

CS was involved in conceiving the study design, conducted data capture from clinical and laboratory records, analysed data and wrote the first draft of the manuscript. AB accessed data from monthly reports of referring ART clinics, conducted statistical analysis and reviewed the manuscript. DS was involved in conception of study design, data capturing, and review of manuscript. MO was involved in accessing data from monthly reports of referring ART clinics, data analysis, and review of manuscript. KR provided clinical support and advice and reviewed manuscript. GM was responsible for conception of study design, supervised the study and reviewed the manuscript. All authors have read and approved the final manuscript.

## Authors' Information

CS is a full-time clinician involved in clinical research, working in the Infectious Diseases Unit at GF Jooste Hospital from 2006. DS is a full-time clinician in the public sector in South Africa. AB and MO are both epidemiologists involved in data management of the Western Cape provincial ART programme. KR is an infectious diseases specialist. GM is an infectious diseases specialist and researcher.
